# Antibiotic Producing Potentials of Three Freshwater Actinomycetes Isolated from the Eastern Cape Province of South Africa

**DOI:** 10.3390/ijms11072612

**Published:** 2010-07-02

**Authors:** Timothy Sibanda, Leonard V. Mabinya, Ntsikelelo Mazomba, David A. Akinpelu, Kim Bernard, Ademola O. Olaniran, Anthony I. Okoh

**Affiliations:** 1 Applied and Environmental Microbiology Research Group (AEMREG), Department of Biochemistry and Microbiology, University of Fort Hare, Alice, South Africa; E-Mails: 200904877@ufh.ac.za (T.S.); lmabinya@ufh.ac.za (L.V.M.); nmazomba@ufh.ac.za (N.M.); 2 Department of Microbiology, Obafemi Awolowo University, Ile-Ife, Nigeria; E-Mail: dakinpel@yahoo.com; 3 South African Environmental Observation Network (SAEON), Elwandle Node, Grahamstown, South Africa; E-Mail: kim@saeon.ac.za; 4 Division of Microbiology, University of KwaZulu Natal, Durban Westville, South Africa; E-Mail: olanirana@ukzn.ac.za

**Keywords:** actinomycetes, crude extract, antibacterial activity, time-kill

## Abstract

Crude extracts of three actinomycetes species belonging to *Saccharopolyspora* (TR 046 and TR 039) and *Actinosynnema* (TR 024) genera were screened for antibacterial activities against a panel of several bacterial strains. The extracts showed antibacterial activities against both gram-negative and gram-positive test bacteria with inhibition zones ranging from 8 to 28 mm (TR 046); 8 to15 mm (TR 039); and 10 to 13 mm (TR 024). The minimum inhibitory concentrations ranged from 0.078 to 10 mg/mL (TR 046); 5 to >10 mg/mL (TR 039); and 1.25 to 5 mg/mL (TR 024). Time-kill studies revealed that crude extract of TR 046 showed strong bactericidal activity against *Bacillus pumilus* (ATCC14884), reducing the bacterial load by 10^4^ cfu/mL and 10^2^ cfu/mL at 4× MIC and 2× MIC, respectively, after 6 h of exposure. Similarly, against *Proteus vulgaris* (CSIR 0030), crude extract of TR 046 achieved a 0.9log_10_ and 0.13log_10_ cfu/mL reduction at 5 mg/mL (4× MIC) and 1.25 mg/mL (2× MIC) after 12 h of exposure. The extract was however weakly bactericidal against two environmental bacterial strains (*Klebsiella pneumoniae* and *Staphylococcus epidermidis*); and against *Pseudomonas aeruginosa* (ATCC 19582): the extract showed bacteriostatic activities at all concentrations tested. These freshwater actinomycetes appear to have immense potential as a source of new antibacterial compound(s).

## 1. Introduction

Actinomycetes are Gram-positive bacteria, which are noteworthy for their antibiotic production, producing more than 70% of all currently known antibiotics [1,2]. The antibiotic substances they produce display antibacterial, antifungal, antitumor, antiprotozoic and antiviral properties [3]. These natural antibiotics have been shaped by evolution to make them effective in killing microorganisms as a competitive tool. Antibiotic production is often associated with sites of high nutrient content as in areas rich in decaying organic matter, with antibiotic production evolving in response to selective pressures created through increased competition [4], and this has been suggested to be related to increased biological activities among actinomycetes associated with detritus in aquatic environments as well as with sediments [5].

Actinomycetes generally have major socioeconomic importance, which include human pathogens such as *Actinomyces israelii* [6,7], non-pathogenic strains which play essential roles as decomposers in terrestrial systems, and antibiotic producers like *Streptomyces* that produce commercially important antibiotics [8] and an array of other secondary metabolites. Antibiotics produced by actinomycetes are normally composed of heterogeneous and biologically active compounds [9].

The antibiotic era is threatened by the relentless rise of resistance in Gram-positive bacterial infections. Much effort is being directed towards developing new compounds to overcome this problem [10]. Although considerable progress is being made within the fields of chemical synthesis and engineered biosynthesis of antimicrobial compounds, nature and actinomycetes in particular, still remain the richest and the most versatile source of new antibiotics [11] of improved efficacies. In this study we investigate antibiotic production by three freshwater actinomycete strains which were isolated from the Tyume River in the Eastern Cape Province of South Africa and identified to belong to the *Saccharopolyspora* (TR 046 and TR 034) and *Actinosynnema* (TR 024) genera [12]. *Actinosynnema* is an aerobic actinomycete that was first isolated from a grass (*Carex* species) blade in Shiga Prefecture, Japan, in September 1976 [13] and is a documented producer of ansamitocins (potent anti-tumor antibiotics). The genus *Saccharopolyspora* was named in 1975 by Lacey and Goodfellow [14]. The most extensively studied of this genus is *Saccharopolyspora erythraea* which is an industrial producer of the antibiotic erythromycin.

In recent years there has been a growing awareness of the potential value of freshwater habitats as a source of actinomycetes that produce secondary metabolites of clinical importance [15]. Aquatic microbes are particularly attractive because they have not been as extensively exploited as their terrestrial counterparts, and because of the high potency required for bioactive compounds to be effective in the aquatic environment, due to the diluting effect of water [16]. A review of literature reveals that little is known concerning the actinomycetes exhibiting antimicrobial properties from this habitat. The list of novel actinomycetes and products derived from poorly explored areas of the world stresses the importance of investigating new habitats [17]. In this paper, we report on the antibiotic production potential of three actinomycetes isolated from a freshwater environment in the Eastern Cape Province of South Africa as part of our exploration for new antimicrobial agents.

## 2. Results and Discussion

### 2.1. Results

When the crude extracts of each of the test actinomycetes were assessed against a total of 32 test bacteria (seven Gram-positive and 25 Gram-negative) at a concentration of 10 mg/mL, extract obtained from *Saccharopolyspora* (TR 046) was observed to be active against nine test bacteria with zones of inhibition ranging from 8–28 mm (Table 1). Extract obtained from *Saccharopolyspora* (TR 039) was active against eight of the test bacteria with zones of inhibition varying from 8–15 mm; while extract obtained from *Actinosynnema* (TR 024) was active against four test bacteria with zones of inhibition ranging from 10–13 mm. Extracts from *Actinosynnema* and *Saccharopolyspora* (TR 039) appeared to be active against only Gram-negative bacteria while extract from *Saccharopolyspora* (TR 046) had a broad spectrum antibacterial activity. *Proteus vulgaris* CSIR 0030 was most susceptible to the extract obtained from *Saccharopolyspora* (TR 046) while the environmental *Staphylococcus epidermidis* was least susceptible. However, with extract from *Saccharopolyspora* (TR 039), *Enterobacter cloacae* ATCC 13047 was least susceptible, while *Proteus vulgaris* ATCC 6830 was most susceptible. There does not appear to be significant dynamics in the susceptibilities of the bacteria for which the extract from *Actinosynnema* was active.

It was also observed that the referenced strains *Pseudomonas aeruginosa* ATCC 7700 and *Proteus* vulgaris CSIR 0030, and environmental strain *Klebsiella pneumoniae* were reactive to all three extracts. Results of the minimum inhibitory concentration (MIC) and minimum bactericidal concentration (MBC) of the test actinomycetes antibiotics are shown in Table 2. The MIC of extract from *Saccharopolyspora* (TR 046) ranged from 0.078 mg/mL to 10 mg/mL while its MBC varied between 1.25 mg/mL and >10 mg/mL. For the extract from *Saccharopolyspora* (TR 039), MIC ranged between 5 and >10 mg/mL while its MBC was generally >10 mg/mL. The extract from *Actinosynnema* on the other hand had MICs ranging between 1.25 mg/mL and 5 mg/mL and an MBC of >10 mg/mL.

Time-kill studies revealed that the *Saccharopolyspora* (TR 046) extract had bacteriostatic effects on referenced bacterial strain *Pseudomonas aeruginosa* (ATCC19582) with no major changes in the bacterial load with time (Table 3). The extract was however, strongly bactericidal against another referenced bacterial strain (*Bacillus pumilus* ATCC 14884) at 4× MIC resulting in the killing of approximately 10^4^ cfu/mL in 6 to 12 h and 10^2^ cfu/mL at 2× MIC after 6 h of exposure to the extract suspension. It was however, weakly bactericidal at normal strength MIC (0.078 mg/mL). The extract also showed good bactericidal activity against the referenced strain *Proteus vulgaris* CSIR 0030 achieving reductions of 0.9log_10_ and 0.13log_10_ cfu/mL at 4× MIC (2.5 mg/mL) and 2× MIC (1.25 mg/mL) respectively after 12 h of exposure. The extract showed limited bactericidal activity against both *Klebsiella pneumoniae* (environmental strain) and *Staphylococcus epidermidis* (environmental strain) at all MIC levels after 6 h of exposure but showed bacteriostatic effects at all MIC levels after 12 h of exposure.

### 2.2. Discussion

When the antibacterial activity of the three freshwater actinomycetes extracts was observed against a series of bacterial isolates, Gram positive test bacteria showed limited susceptibility to the extracts while the Gram negative bacteria were more susceptible. Whilst our findings confirm antibiotic production in the *Saccharopolyspora* and *Actinosynnema* isolated from the freshwater habitat, nevertheless, the *Saccharopolyspora* (both TR 046 and TR 039) species appear to exhibit consistently greater antibacterial activity than *Actinosynnema* (TR 024). The limited antibacterial activity of *Actinosynnema* extract is in agreement with previous findings [13] which reported that actinomycetes belonging to the genus *Actinosynnem*a mainly produce maytansinoid antibiotics (potent anti-tumor agents) although it is worth noting that they still do produce antibacterials, howbeit against a limited range of bacteria, whereas *Saccharopolyspora* is a well documented producer of antibiotics [18], with *Saccharopolyspora erythraea* well known for the industrial production of the antibiotic erythromycin.

Of the two *Saccharopolyspora* strains (TR 046 and TR 039), extract obtained from TR 046 produced consistently larger inhibition zone diameters than that of TR 039. This result suggests that the extract from TR 046 has greater efficacy compared to the extract from TR 039. This result is further proven by the MIC and MBC results which show that for the extract from TR 039 MIC values ranged from 5 mg/mL to >10 mg/mL while the MBC values were all >10 mg/mL. The observed differences in the antibiotic producing potentials of the two *Saccharopolyspora* strains, TR 046 and TR 039, can possibly be explained in terms of the ecological conditions from where they were isolated. Actinomycetes isolated from a habitat richer in microorganism (competing bacterial species) tend to produce more antagonistic substances as a competitive tool for survival compared to those isolated from places with low numbers of competing microorganisms [19]. In this regard, it can be suggested that *Saccharopolyspora* TR 046 was isolated from a habitat richer in competing species of microorganisms compared to *Saccharopolyspora* TR 039.

Extracts obtained from both *Saccharopolyspora* TR 046 and TR 039 were active against five referenced bacterial strains each and whereas the extract from TR 046 was active against three environmental strains and showed no activity against clinical isolates, the extract from TR 039 showed activity against two environmental strains and one clinical isolate, a result which emphasizes the similarities in the genotypic origin of the two organisms. Their limited activity against clinical isolates confirms that these clinical strains of bacteria may possess characteristics that differ or are absent from the non-pathogenic strains [20]. Pathogenic strains may possess specific virulence determinants (toxins and adhesions, *etc.*) encoded by monocistronic genes, plasmids, or pathogenicity islands as well as plasmids that code for drug resistance [21,22], which may partially account for the ineffectiveness of the extracts against clinical strains.

Our findings suggest limited activity against Gram-positive bacteria by all the actinomycetes extracts used although it is worth noting that seven Gram-positive bacteria were tested as compared to 25 Gram-negative test bacteria and it can therefore be deduced that it may not be necessarily correct to suggest that the extracts lacked broad spectrum activity. Besides, Gram-negative bacteria are inherently more resistant to antimicrobials than Gram-positive bacteria due to the combined exclusion of the antimicrobial compounds by the double membrane barrier and transmembrane efflux present in this group of organisms [23], hence sourcing actinomycetes that produce effective antimicrobials against Gram-negative bacteria is a step in the right direction for the war against antibiotic resistance.

The rate of kill of the test organisms by the extract from *Saccharopolyspora* TR 046 appears to be both concentration and time dependent. Results suggest that the extract was mostly bactericidal at 2× MIC and 4× MIC up to 6 h of exposure with the effect waning off after 6 h. This might suggest that dosing frequency needs to be increased to once after every 6 h to maintain the bactericidal effect of the extract at its optimum. Complete eradication of the test organisms was not achieved. In view of the knowledge that actinomycetes produce secondary metabolites, especially antibiotics, in order to overcome other competing microorganisms by killing them [19], this could be attributed to the fact that the sediments from which the actinomycetes isolates were obtained have a small bacterial population which competes with them and hence rely less on antibiotic production as a competitive tool against other bacteria.

However, the extract exhibited a strong bactericidal efficacy against *Bacillus pumilus* (ATCC 14884) achieving a 1.26log_10_ reduction in counts of the test organism after 6 h of exposure at 0.312 mg/mL (4× MIC) and moderate bactericidal efficacy against *Proteus vulgaris* CSIR 0030 achieving a 0.9log_10_ reduction in counts of the test organism after 12 h of exposure at 2.5 mg/mL (4× MIC). The extract exhibited bacteriostatic effects on two test bacteria (*Klebsiella pneumoniae* {KZN} and *Staphylococcus epidermidis* {KZN}) with no major changes on the bacterial load with time. *Pseudomonas aeruginosa* showed persistive response to the extract, with a marked delay in its growth at 2.5 mg/mL (4× MIC) after 6 h of exposure increasing by 0.01log_10_ counts as compared to an increase of 1.02log_10_ counts after 6 h of exposure at 0.625 mg/mL (MIC). A 3log_10_ or 99.9% reduction in viable bacterial density in an 18–24 h period is the generally accepted definition of bactericidal activity in antibiotics [24].

### 2.3. Statistical Analysis

Results were analyzed under Minitab Release 14.2 using a 2-sample T-test and One-way Analysis Of Variance (ANOVA) as the statistical packages. Mean zone diameters were compared for all the three actinomycetes extracts. Analysis was carried out at 95% confidence interval.

## 3. Methods and Materials

### 3.1. Test Actinomycetes

Three actinomycetes strains belonging to the genera *Saccharopolyspora* (TR 046 and TR 039) and *Actinosynnema* (TR 024) were obtained from the culture collections of the Applied & Environmental Microbiology Research Group (AEMREG), University of Fort Hare, Alice, South Africa. The strains were isolated from the Tyume River in the Eastern Cape Province of South Africa [12]. The organisms were maintained on agar slants and in 20% glycerol (at −80 °C).

### 3.2. Preparation of Actinomycetes Inocula

The stock culture of the test actinomycetes used in this study were prepared by streaking the actinomycetes from the agar slants onto starch casein agar (SCA) which was prepared as follows (per liter of filtered freshwater): soluble starch, 10 g; potassium phosphate dibasic, 2 g; potassium nitrate, 2 g; sodium chloride, 2 g; casein, 0.3 g; magnesium sulfate.7H_2_O, 0.05 g; calcium carbonate, 0.002 g; ferrous sulfate.7H_2_O, 0.01 g and bacteriological agar, 16 g. Each of these compounds was added to the diluents and allowed to dissolve completely using a magnetic stirrer. The medium was then autoclaved at 121 °C and 15 mm Hg for 15 minutes and allowed to cool down to 50 °C before being poured into 90 mm Petri dishes. The test actinomycetes were streaked on the prepared medium and incubated at 28 °C for between 7 and 14 days under aerobic conditions. Actinomycetes inocula were then prepared by transferring several colonies into sterile normal saline (10 mL) and the suspensions vortexed for 20 seconds to ensure homogeneity.

### 3.3. Preparation and Inoculation of Fermentation Broth

The fermentation broth was prepared following a method outlined by Muiru *et al.* [25] as follows (per liter of distilled water): 10 g starch, 4 g yeast extract, 2 g peptone, 5 ml potassium bromide (20 g/L) and 5 mL iron (iii) sulfate tetrahydrate (4.76 g/L). The medium was divided into 500 mL aliquots into 1 L Erlenmeyer flasks and sterilized by autoclaving at 121 °C and 15 mm Hg for 15 minutes. After the medium cooled, 100 μL volumes of actinomycetes suspensions (standardized to McFarland 0.5) were used to inoculate the flasks. The flasks were then incubated at 27 °C on a shaker at 300 rpm for 10 days. For quality control, confirmation of purity was done by streaking the fermentation cultures onto nutrient agar (NA), potato dextrose agar (PDA) and starch casein agar (SCA) plates.

### 3.4. Extraction of the Crude-Antibiotic Extracts from Fermentation Cultures

Crude antibiotic extracts were recovered from the broth culture filtrate by solvent extraction using ethyl acetate in accordance with the description of Liu *et al.* [26]. Ethyl acetate was added to the filtrate in the ratio 1:1 (v/v) and shaken vigorously for 1 h for complete extraction. The ethyl acetate phase that contained the antibiotic was separated from the aqueous phase and concentrated *in vacuo* at 60 °C using a rotary evaporator. The residue obtained was weighed and reconstituted in 50% methanol to make a working concentration of 10 mg/mL for the antibacterial assays.

### 3.5. Test Bacteria and Inocula Preparation

The test bacteria used in this study were obtained from the culture collection of the Applied and Environmental Microbiology Research Group (AEMREG) laboratory at the University of Fort Hare, Alice, South Africa and included the following:

**Referenced strains:** *Escherichia coli* ATCC 8739, *Pseudomonas aeruginosa* ATCC 19582, *Staphylococcus aureus* ATCC 6538, *Enterococcus faecalis* ATCC 29212, *Bacillus cereus* ATCC 10702, *Bacillus pumilus* ATCC 14884, *Pseudomonas aeruginosa* ATCC 7700, *Enterobacter cloacae* ATCC 13047, *Klebsiella pneumoniae* ATCC 10031, *Klebsiella pneumoniae* ATCC 4352, *Proteus vulgaris* ATCC 6830, *Proteus vulgaris* CSIR 0030, *Serratia marcescens* ATCC 9986, *Acinetobacter calcoaceticus, Acinetobacter calcoaceticus anitratus, Escherichia coli* 25922.
**Environmental strains:** *Klebsiella pneumoniae, Bacillus subtilis, Shigella dysenteriae, Staphylococcus epidermidis, Pseudomonas aeruginosa, Proteus vulgaris, Enterococcus faecalis, Staphylococcus aureus, Micrococcus kristinae* and *Micrococcus luteus, Shigella flexineri, Salmonella sp*.
**Clinical isolates:** *Staphylococcus aureus* OKOH 1, *Staphylococcus aureus* OKOH 2A, *Staphylococcus aureus* OKOH 3, and *Staphylococcus sciuri* OKOH 2B.

The test bacteria were confirmed for purity by streaking onto nutrient agar plates. These pure bacterial isolates were then inoculated into nutrient broth and incubated at 37 °C for 24 h. The turbid broths were later centrifuged at 7000 rpm and the supernatant discarded. The pellets of cells were resuspended and double washed in sterile normal saline and standardized to OD_600nm_ 0.1. The washed and standardized cells were subsequently used for various experiments described below.

### 3.6. Antibacterial Susceptibility Tests

Antibacterial activities of the crude extracts were determined using agar well diffusion technique as described by Pandey *et al.* [27]. Test organism cultures were grown overnight (18 h) in nutrient broth and standardized to OD_600nm_ 0.1. Test organisms were then spread-plated onto Muller Hinton agar (MHA) plates using sterile cotton swabs. A flame sterilized cork borer with a diameter of 6 mm was used to bore wells into the agar and 100 μL of the extract (10 mg/mL) loaded into the wells. Control wells were loaded with 100 μL of 50% methanol. The extract was allowed to diffuse into the agar before the plates were incubated under aerobic conditions at 37 °C for 24 h. At the end of the incubation period, the plates were observed for zones of inhibition around the wells. Inhibition zone is defined as the area free of growth in a bacterial lawn which results from the effect of antibiotic that has diffused into the medium from its applied source [28].

### 3.7. Determination of the Minimum Inhibitory Concentration (MIC) and Minimum Bactericidal Concentration (MBC)

The MICs were determined using test organisms that showed susceptibility to the crude extracts by the broth microdilution method as outlined by the EUCAST Discussion Document [29]. Sterile plastic, disposable microtiter plates with 96 flat-bottom wells were used. The medium used in the plates was prepared at double the final strength to allow for a 50% dilution once the inoculum and solvents or antimicrobial were added. A 100 μL volume of double strength Muller Hinton broth was introduced into all the 96 wells and varying concentrations of the antibiotic were added in decreasing order along the wells after which wells were loaded with 50 μL of the test organism suspension. The plates were then incubated at 37 °C for 18–24 h. Wells in column 12 were used as the growth controls and contained 50 μL of test organism and 50 μL of sterile distilled water. Results were read using a microtiter plate reader (BIO-RAD model 680) at 490 nm. Visual reading of results was also done by first adding resazurin dye into all the wells. Wells with no growth turned blue in color while those with growth turned pink, and this helped to give a clear visual demarcation of the MIC wells. The MIC was estimated as the lowest concentration of the extract that inhibited growth of the test organisms.

The MBC was determined from the MIC plate following a method outlined by the CLSI [30], and is defined as the lowest concentration of an antibiotic that under defined *in vitro* conditions reduces by 99.9% the number of organisms in a medium containing a defined inoculum of bacteria, within a defined period of time [28]. It was determined by inoculating the broths in the MIC range into drug-free nutrient agar medium. The MBC was determined as the antibiotic concentration at which no growth was observed after incubation for 48 h.

### 3.8. Determination of the Rate of Kill of the Crude Extract

The rate of kill assay was done only for TR 046 extract, which appeared to exhibit more antibacterial potency than the other actinomycetes. This was done by monitoring bacterial cell death over time in accordance with the description of Okoli and Iroegbu [31]. Also, five test bacteria were selected for this assay based on their susceptibility and Gram’s reaction. The inocula were prepared following the described guidelines of the EUCAST Discussion Document [29]. The resultant cell suspension was diluted 1:100 with fresh sterile broth and used to inoculate 50 mL volume of nutrient broth incorporated with the extract at multiples of the MIC to a final cell density of 5 × 10^5^ cfu/mL [32,33]. The flasks were then incubated with shaking at 37 °C on an orbital shaker at 120 rpm and samples of 100 μL were then withdrawn at 6 h and 12 h intervals and diluted appropriately. Approximately 100 μL volumes of the diluted samples were then plated out in triplicate on nutrient agar. Plates were incubated at 37 °C for 24 h, after which the numbers of surviving cells were enumerated [33]. Controls consisted of extract free nutrient broth inoculated with test organism.

## 4. Conclusions

In conclusion, this study has shown that freshwater environments could serve as potential reservoirs for actinomycetes of antimicrobial importance with varying spectra of activities. A detailed characterization of the active principles of the antibacterial extracts is the subject of ongoing investigation in our group.

## Figures and Tables

**Table 1.** Antibacterial activities of crude extracts obtained from *Saccharopolyspora* (TR 046 and TR 039) and *Actinosynnema* (TR024) isolates.

**Test organism**	**Gram reaction**	**Antibacterial activity [mm of inhibition zone diameter]**		
		**A**	**B**	**C**
*Enterococcus faecalis* ATCC 29212*	+	−	−	−
*Bacillus cereus* ATCC 10702*	+	−	−	−
*Bacillus pumilus* ATCC 14884*	+	+ (27)	−	−
*Micrococcus kristinae*™	+	−	−	−
*Bacillus subtilis*™	+	−	−	−
*Micrococcus luteus*™	+	−	−	−
*Staphylococcus epidermidis*™	+	+ (8)	−	−
*Pseudomonas aeruginosa* ATCC 7700*	−	+ (17)	+ (10)	+ (13)
*Enterobacter cloacae* ATCC 13047*	−	−	+ (8)	−
*Klebsiella pneumoniae* ATCC 10031*	−	−	−	−
*K. Pneumoniae* ATCC 4352*	−	−	−	−
*Proteus vulgaris* ATCC 6830*	−	−	+ (15)	+ (13)
*Proteus vulgaris* CSIR 0030*	−	+ (28)	+ (12)	+ (12)
*Serratia marcescens* ATCC 9986*	−	+ (20)	+ (14)	−
*Staphylococcus aureus* ATCC 6538*	−	−	−	−
Acinetobacter calcoaceticus*	−	+ (17)	−	−
Acinetobacter calcoaceticus anitratus*	−	−	−	−
*Klebsiella pneumoniae*™	−	+ (20)	+ (12)	+ (10)
*Escherichia coli* ATCC 8739*	−	−	−	−
*Shigella flexineri*™	−	−	−	−
*Escherichia coli* ATCC 25922*	−	−	−	−
*Salmonella* sp™	−	−	+ (13)	−
*Pseudomonas aeruginosa* ATCC 9582*	−	+ (16)	−	−
*Pseudomonas aeruginosa*™	−	+ (12)	−	−
*Proteus vulgaris*™	−	−	−	−
*Enterobacter faecalis*™	−	−	−	−
*Escherichia coli*™	−	−	−	−
*Staphylococcus aureus*™	−	−	−	−
*Staphylococcus aureus* OKOH 1^®^	−	−	+ (13)	−
*Staphylococcus aureus* OKOH 2a^®^	−	−	−	−
*Staphylococcus aureus* OKOH 2b^®^	−	−	−	−
*Staphylococcus aureus* OKOH 3^®^	−	−	−	−

A = extract obtained from TR 046 (concentration 10 mg/mL).

B = extract obtained from TR 039 (concentration 10 mg/mL).

C = extract obtained from TR 024 (concentration 10 mg/mL).

Diameter of zones of inhibition exclude the perimeter of the well; (−) denotes no activity; (+) denotes activity;

* denotes referenced strains;

™ denotes environmental strains;

® denotes clinical isolates.

**Table 2.** MIC and MBC results for extracts obtained from *Saccharopolyspora* (TR 046 and TR 039) and *Actinosynnema* (TR 024).

**Extract**	**Test organism**	**Gram**	**MIC**	**MBC**
**TR 046**	*Staphylococcus epidermidis*™	+	0.078	1.25
	*Bacillus pumilus* ATCC 14884*	+	0.078	10
	Acinetobacter calcaoceticus*	−	1.25	>10
	*Pseudomonas aeruginosa* ATCC 7700*	−	0.625	5
	*Pseudomonas aeruginosa* ATCC 19582*	−	0.625	2.5
	*Proteus vulgaris* CSIR 0030*	−	0.625	5
	*Serratia marcescens* ATCC 9986*	−	0312	10
	*Klebsiella pneumoniae™*	−	1.25	5
	*Pseudomonas aeruginosa™*	−	10	>10

**TR 039**	*Pseudomonas aeruginosa* ATCC 7700*	−	>10	>10
	*Enterobacter cloacae* ATCC 13047*	−	>10	>10
	*Proteus vulgaris* ATCC 6830*	−	10	>10
	*Proteus vulgaris* CSIR 0030*	−	5	>10
	*Serratia marscens* ATCC 9986*	−	5	>10
	*Klebsiella pneumoniae*™	−	>10	>10
	*Salmonella* spp™	−	5	>10
	*Staphylococcus aureus* OKOH1^®^	−	5	>10

**TR 024**	*Pseudomonas aeruginosa* ATCC 7700*	−	5	>10
	*Proteus vulgaris* ATCC 6830*	−	5	>10
	*Proteus vulgaris* CSIR 0030*	−	1.25	>10
	*Klebsiella pneumoniae™*	−	1.25	>10

* denotes referenced strain;

™ denotes environmental strain;

® denotes clinical isolate;

MIC = minimum inhibitory concentration; MBC = minimum bactericidal concentration.

**Table 3.** Time kill results for extract obtained from *Saccharopolyspora* (TR 046).

**Susceptible isolate**	**MIC (mg/mL)**			**Log_10_**	**Kill**		
		**4xMIC**		**2xMIC**		**MIC**	
	
		6 h	12 h	6 h	12 h	6 h	12 h
*Bacillus pumilus* ATCC 14884*	0.078	1.26	0.31	1.13	0.12	0.20	−2.41
*Klebsiella pneumoniae*™	1.25	0.26	−0.05	0.25	−1.00	0.77	−0.27
*Pseudomonas aeruginosa* ATCC 19582*	0.625	−0.01	−0.30	−0.09	−0.12	−0.92	−1.31
*Staphylococcus epidermidis*™	0.078	0.74	−0.19	0.54	−0.27	0.02	−0.45
*Proteus vulgaris* CSIR 0030*	0.625	0.09	0.90	0.08	0.13	−0.11	−0.04

* denotes referenced strain;

™ denotes environmental strain;

(−) denotes bacteriostatic effect;

MIC = minimum inhibitory concentration.
